# Phosphoproteomic Analysis of Gossypol-Induced Apoptosis in Ovarian Cancer Cell Line, HOC1a

**DOI:** 10.1155/2014/123482

**Published:** 2014-08-12

**Authors:** Lixu Jin, Yuling Chen, Xinlin Mu, Qingquan Lian, Haiyun Deng, Renshan Ge

**Affiliations:** ^1^The First Affiliated Hospital of Wenzhou Medical University, Wenzhou, Zhejiang 325000, China; ^2^School of Life Sciences, Tsinghua University, Beijing 100084, China; ^3^Peking University People's Hospital, Beijing 100044, China; ^4^The Second Affiliated Hospital of Wenzhou Medical University, Wenzhou, Zhejiang 325027, China; ^5^Institute of Reproductive Biomedicine, Wenzhou Medical University, Wenzhou, Zhejiang 325027, China

## Abstract

Ovarian cancer is a major cause for death of gynecological cancer patients. The efficacy of traditional surgery and chemotherapy is rather compromised and platinum-resistant cancer recurs. Finding new therapeutic targets is urgently needed to increase the survival rate and to improve life quality of patients with ovarian cancer. In the present work, phosphoproteomic analysis was carried out on untreated and gossypol-treated ovarian cancer cell line, HOC1a. We identified approximately 9750 phosphopeptides from 3030 phosphoproteins, which are involved in diverse cellular processes including cytoskeletal organization, RNA and nucleotide binding, and cell cycle regulation. Upon gossypol treatment, changes in phosphorylation of twenty-nine proteins including YAP1 and AKAP12 were characterized. Western blotting and qPCR analysis were used to determine expression levels of proteins in YAP1-related Hippo pathway showing that gossypol induced upregulation of LATS1, which phosphorylates YAP1 at Ser 61. Furthermore, our data showed that gossypol targets the actin cytoskeletal organization through mediating phosphorylation states of actin-binding proteins. Taken together, our data provide valuable information to understand effects of gossypol on protein phosphorylation and apoptosis of ovarian cancer cells.

## 1. Introduction

Ovarian cancer is a major cause for death of gynecological cancer patients. Early diagnosis of ovarian cancer is difficult, while its progression is fast. The standard treatment is surgical removal followed by platinum-taxane chemotherapy. However, the efficacy of traditional surgery and chemotherapy is rather compromised and platinum-resistant cancer recurs in approximately 25% of patients within six months, resulting in 31% overall five-year survival rate [1–3]. Virtually no efficient second-line treatment is available. Therefore, a deep understanding of biology of ovarian cancer and finding new therapeutic targets are urgently needed to increase the survival rate and to improve life quality of patients with ovarian cancer.

Genomic analysis has shown that ovarian cancer is characterized by TP53 mutations in almost all tumors and by germline mutations in BRCA1/2 in approximately 10% of high-grade serous ovarian adenocarcinoma (HGS-OvCa) [4]. The Bcl-2 is a 26 kDa integral membrane oncoprotein localized in the inner mitochondrial and cell membrane. The major function of Bcl-2 is to inhibit apoptosis by arresting cells in the G0/G1 phase of cell cycle. It has been reported that Bcl-2 proteins are overexpressed in many different solid tumors such as breast, prostate, and lung cancer and that serum Bcl-2 levels are also significantly higher in patients with ovarian cancer [5, 6]. The structure of Bcl-2 has BH3 domains, known as the BH3 binding groove, into which Bad, Bid, and Bim bind [7–9]. The binding groove in Bcl-2 and Bcl-xL proteins is essential for their antiapoptotic function. The small molecules binding to the BH3 binding groove of Bcl-2/Bcl-xL block the heterodimerization of Bcl-2 with proapoptotic members of the Bcl-2 protein family, leading to apoptosis of cancer cells [10–12].

Gossypol is a phenolic aldehyde extracted from the cotton and the tropical plants. Gossypol forms an extensive hydrogen bonding network with residues Arg146 and Asn143 in Bcl-2 with the aldehyde group and the adjacent hydroxyl group on the right naphthalene ring [13]. Gossypol has been identified as a BH3-mimetic inhibitor of proapoptotic Bcl-2 family members, including Bcl-2, Bcl-xL, and Mcl-1, to induce apoptosis in cancer [14–17]. Gossypol is now in phase II and IIb clinical trials for hormone-refractory prostate cancer with promising initial results [18, 19]. Gossypol and its derivatives have been found to act as an inhibitor for several dehydrogenase enzymes [20, 21] by binding to lactate dehydrogenase and inhibiting its activity. Gossypol also inhibits growth of prostate cancer cells by modulation of TGF beta/Akt signaling [22], activates p53 to induce apoptosis in prostate cancer cells [23], and enhances radiation-induced apoptosis through SAPK/JNK pathway [24]. On the other hand, gossypol and ApoG2 suppress the c-Myc signaling pathway [25] and NF-kappaB activity by decreasing NF-kappaB-related gene expression in human leukemia U937 cells [26]. Furthermore, gossypol affects ROS-dependent mitochondria by activating death receptor 5 pathway in human colorectal carcinoma cells [27–29]. It also reversibly inhibits calcineurin and binds to calmodulin [30]. Recent studies have shown that gossypol and its enantiomer (AT-101) regulate expressions of proangiogenic molecules released from cancer cells, suggesting the potential role of gossypol in antiangiogenesis by modulating VEGF signaling-mediated angiogenesis [31, 32]. Despite its involvement in signaling pathways, global phosphoproteomic analysis of gossypol-treated cancer cells has, however, not been performed that is important to identify the new gossypol-mediated cellular process and new therapeutic targets.

Protein phosphorylation is involved in diverse signaling pathways in cells and regulates a series of biological processes including cell growth, differentiation, proliferation, apoptosis, and even intercellular communication [33, 34]. Phosphorylation is the key process in tumor initiation and progression. Aberrant phosphorylation has been frequently linked to ovarian cancer. For example, activation of AKT2 kinase and PIK3CA has been found in ovarian cancer carcinogenesis [35–37]. EGFR has been found in 33–75% of ovarian cancers and has been implicated for growth and progression of ovarian cancer. Signal transducer and activator of transcription 3 has been shown to be constitutively active in ovarian carcinoma cells and in drug-resistant ovarian cancer [38–41]. Proteomic methods have been developed over the last several years to characterize protein phosphorylation, including enrichment of low-abundance phosphoproteins or phosphopeptides with immobilized metal affinity chromatography (IMAC), strong cation exchange chromatography (SCX), or the two in combination [42, 43]. Titanium dioxide (TiO_2_) particles have been shown as an efficient affinity matrix to enrich phosphopeptides from a complex mixture [44].

In the present work, we have employed TiO_2_ particles as an affinity matrix to enrich phosphopeptides from ovarian cancer cell line, HOC1a, which was developed in Beijing University Hospital. HOC1a has been used as the model system to study the immunotherapy of ovarian cancers. 9750 phosphoproteins were identified in HOC1a cells, and gossypol treatment resulted in changes in phosphorylation of 29 phosphoproteins including YAP1 and Ras GTPase-activating protein-binding protein 1. Our data indicate that gossypol modulates phosphorylation states of YAP1 and other actin-binding proteins that may affect cell migration and cell viability.

## 2. Materials and Methods

### 2.1. Chemicals and Reagents

Medium and serum were purchased from Wistent (Saint-Jean-Baptiste, CA) and used without further purification. Dithiothreitol (DTT) and iodoacetamide (IAA) were purchased from Sigma (St. Louis, MO). Sequence grade modified trypsin was purchased from Promega (Fitchburg, WI). TiO_2_ tips were from GL Sciences (Tokyo, Japan).

### 2.2. Cell Culture and Sample Preparation

The HOC1a cell line was obtained from Peking University People's Hospital (Beijing, China). Cells were cultured in DMEM supplemented with 15% fetal bovine serum (FBS), 1% penicillin-streptomycin (Wistent, Saint-Jean-Baptiste, CA) in 150 mm plates (Corning, New York, USA) and maintained at 37°C in a humidified atmosphere with 5% CO_2_. Growth and morphology were continually monitored, and cells were passaged when they had reached 90% confluence. For gossypol treatment, 10 *μ*M gossypol dissolved in dimethyl sulfoxide (DMSO) was added into HOC1a cells for a specific time. Cells were lysed in a buffer containing 1% Nonidet P-40, 0.1% sodium deoxycholate, 150 mM NaCl, 1 mM EDTA, 50 mM Tris, pH 7.5, 1 mM sodium orthovanadate, 5 mM NaF, 5 mM beta-glycerophosphate, and protease inhibitors (Complete Tablet, Roche Diagnostics). Lysates were centrifuged at 17,000 ×g for 15 min to pellet cellular debris. The supernatant was collected.

### 2.3. Analysis of Apoptosis by Propidium Iodide Staining

Briefly, untreated and gossypol-treated HOC1a cells were trypsinized, washed with ice-cold 0.9% PBS, and then fixed in precooled 70% ethanol at 4°C for 1.5 h, respectively. After fixation, cells were washed with 0.9% PBS and resuspended in 0.5 mL PBS containing 250 *μ*g/mL RNase A at 37°C for 30 min. Cells were then incubated with 100 *μ*g/mL propidium iodide (PI) in the dark at 4°C for 30 min before being transferred to FACS tubes for flow cytometry analysis. DNA content was analyzed using BD FACSCalibur (Becton Dickinson, Franklin Lakes, USA) and data were acquired and analyzed using CellQuest software (Becton Dickinson).

### 2.4. Enrichment of Phosphopeptides

Proteins were precipitated by addition of four volumes of methanol, one volume of chloroform, and three volumes of water in a sequential manner. The addition of each solvent was followed by a short vortex. After centrifugation at 17,000 ×g for 1 min, proteins were focused between organic and inorganic phases. The aqueous phase was discarded. Four starting volumes of methanol were then added to the protein pellet followed by a short vortex. After spinning down at 17,000 ×g for 2 min, methanol was removed, and the protein pellet was air-dried. Precipitated proteins were redissolved in a buffer containing 6 M urea, 2 M thiourea, and 50 mM ammonium bicarbonate, pH 7.5. Protein concentrations were determined using a Bradford method. Equal amounts of proteins from untreated and gossypol-treated HOC1a cells were used for phosphoproteomic analysis. Proteins were reduced with 1 mM dithiothreitol for 1 h and alkylated with 5.5 mM iodoacetamide for 45 min in the dark. After diluting for four times with 50 mM ammonium bicarbonate, samples were digested overnight with sequencing grade modified trypsin (1/50 w/w). The digestion was quenched by adding trifluoroacetic acid. Peptides were desalted with Waters Oasis HLB 1cc column.

The digested peptides were mixed with 100 *μ*L binding buffer (0.3% TFA, 60% acetonitrile, and 25% lactic acid) and loaded on a TiO_2_ tip. After successive washing with washing buffer (0.4% TFA, 80% acetonitrile), an elution buffer containing 5% ammonium hydroxide and 5% pyrrolidine was used to elute phosphopeptides. Eluted peptides were acidified with TFA and desalted using C18 StageTips followed by LC-MS/MS analysis.

### 2.5. Characterization of Phosphorylation Sites of Proteins in HOC1a Cells

For LC-MS/MS analysis, phosphopeptides were separated by a 100 min gradient elution at a flow rate 0.250 *μ*L/min with the EASY-nLCII integrated nano-HPLC system (Proxeon, Denmark) which was directly interfaced with a Thermo LTQ-Orbitrap mass spectrometer. The analytical column was a homemade fused silica capillary column (75 *μ*m ID, 150 mm length; Upchurch, Oak Harbor, WA) packed with C-18 resin (300 A, 5 *μ*m; Varian, Lexington, MA). Mobile phase A consisted of 0.1% formic acid, and mobile phase B consisted of 100% acetonitrile and 0.1% formic acid. An LTQ-Orbitrap mass spectrometer was operated in the data-dependent acquisition mode using Xcalibur 2.0.7 software and there is a single full-scan mass spectrum in the Orbitrap (400–1800* m/z*, 30,000 resolution) followed by 20 data-dependent MS/MS scans in an ion trap at 35% normalized collision energy (CID).

MS/MS spectra from each LC-MS/MS run were searched against the IPI human database using an in-house Mascot or Proteome Discoverer (Version 1.3) searching algorithm. The search criteria were as follows: full tryptic specificity was required; two missed cleavages were allowed; carbamidomethylation was set as fixed modification; oxidation (M) and phosphorylation (STY) were set as variable modifications; precursor ion mass tolerance was 10 ppm for all MS acquired in the Orbitrap mass analyzer; and fragment ion mass tolerance was 0.5 Da for all MS2 spectra acquired in the LTQ. High confidence score filter (FDR < 1%) was used to select the “hit” peptides and their corresponding MS/MS spectra were manually inspected. Phosphorylation sites were determined by the PhosphoRS algorithm, which calculates the site probability to estimate that the site is truly phosphorylated. When the calculated pRS probability is above 75%, the site assignment is considered to be the true hit. When a phosphopeptide was identified in more than three different experiments, label-free quantitation was carried out to determine its relative changes before and after gossypol treatment using the extracted ion current.

### 2.6. Western Blotting

HOC1a cells were treated with 10 *μ*M gossypol for 4 h. The control and gossypol-treated cells were lysed with RIPA lysis buffer (50 mM Tris-HCl, 150 mM NaCl, 0.1% SDS, 1% NP-40, 0.5% sodium deoxycholate, 1 mM PMSF, protease inhibitors, and phosphatase inhibitors l from Solabio (Beijing, China)) for 30 min on ice. The supernatant was collected after centrifugation for 30 min at 4°C. Protein concentrations were determined using a BCA assay. Proteins were separated by 1D SDS-PAGE and transferred onto a PVDF membrane by electroblotting. After blocking with 5% nonfat milk for 2 h at room temperature, the membrane was incubated overnight at 4°C with 2000-fold diluted primary antibody (rabbit anti-YAP1, from Abcam, UK), washed with TBST buffer for 3 times (each 10 minutes), and then incubated for 2 h with 2000-fold diluted anti-rabbit secondary antibody labeled with HRP. After washing with TBST buffer for 3 times (each 10 min), images were developed using Enlight western blotting detection reagents from Engreen Biosystem (Beijing, China). *β*-Actin was detected with anti-*β*-actin antibodies as an internal control.

### 2.7. Quantitative Real-Time PCR (qPCR)

HOC1a cells were seeded in 100 mm plate (1 × 10^6^ cells each) for 24 h and treated with 10 *μ*M gossypol for an additional 4 hours. The total RNA was extracted from each sample using Trizol reagent (Invitrogen, Carlsbad, CA) according to the manufacturer's instructions. RNA quality was examined by agarose gel electrophoresis. Concentrations of RNA were determined using ultraviolet photometry and were immediately transcribed into cDNA using Tiangen 1st strand cDNA synthesis kit from Tiangen (Beijing, China). Specific PCR primers were designed using the Primer-Blast tool (http://www.ncbi.nlm.nih.gov/tools/primer-blast/). These primers are listed in Supplementary Table 1 available online at http://dx.doi.org/10.1155/2014/123482. Quantitative real-time PCR was performed using SYBR green master mix (Tiangen, Beijing, China) on Roche 480 instrument (Roche, Rotkreuz, Switzerland). Amplification was performed using the following cycles: denaturation at 94°C, 2 min; amplification at 94°C for 15 s, 55°C for 15 s, and 68°C for 15 s with 40 cycles; followed by a final extension at 72°C for 10 min. *β*-Actin was used as internal control. The ratio of mRNA level was showed as 2^−ΔΔCT^. The experiment was repeated three times.

### 2.8. Statistical Analysis

To compare two groups, Student's *t*-test was used. Differences with *P* < 0.05 were considered statistically significant.

## 3. Results

### 3.1. Gossypol-Induced Apoptosis of HOC1a Cells

**Figure 1:**
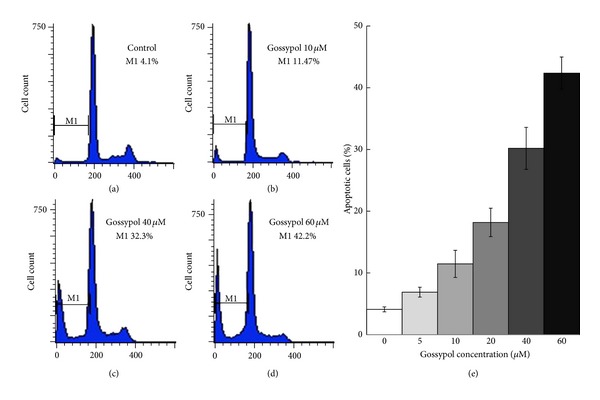
FACS analysis of gossypol-induced apoptosis in HOC1a cells. The histogram of PI staining for (a) control; (b) 10 *μ*M gossypol-treated cells for 24 hours; (c) 40 *μ*M gossypol-treated cells for 24 hours; and (d) 60 *μ*M gossypol-treated cells for 24 hours; (e) percentage of apoptosis related cell death in HOC1a cells treated with gossypol at different concentrations for 24 hours. Results are expressed as the mean of three experiments. Significant apoptosis was induced.

HOC1a cells were treated with gossypol at different concentrations for different periods of time. FACS analysis was performed to determine apoptosis and DNA fragmentation in gossypol-treated HOC1a cells by propidium iodide staining. Gossypol treatment increases the cell population in the M1 area compared to untreated cells. Concomitantly, the population of healthy cells was decreased. The quantitative FACS data representing the percentage of live and apoptotic cells based on DNA fragmentation are shown in Figure 1. We estimated the percentage of apoptotic cells reached 42% after cells were treated with 60 *μ*M gossypol for 24 h.

### 3.2. Phosphoproteomic Analysis of Vehicle Control and Gossypol-Treated HOC1a Cells

In the present study, we focused on changes in phosphorylation of HOC1a cells upon 10 *μ*M gossypol treatment for 4 h. Using the high confidence score filter, we identified 9754 and 9540 phosphopeptides, respectively, in untreated and gossypol-treated cells, leading to identification of 3032 and 3092 phosphoproteins. Experiments were repeated three times and 62 phosphoproteins with higher scores were chosen (Supplementary Table 2) and their phosphorylation sites were further confirmed by Scaffold PTM-Proteome Software. Gene Ontology (GO) was used to categorize the identified phosphoproteins into several significant groups including RNA binding and processing, cytoskeletal organization, and transcription regulation (Supplementary Figure 1). Many of them also play crucial roles in various physiological and pathological processes. For example, Isoform 1 of Bcl-2-associated transcription factor 1 and interferon gamma-inducible protein 16 regulate cell apoptosis.

**Figure 2:**
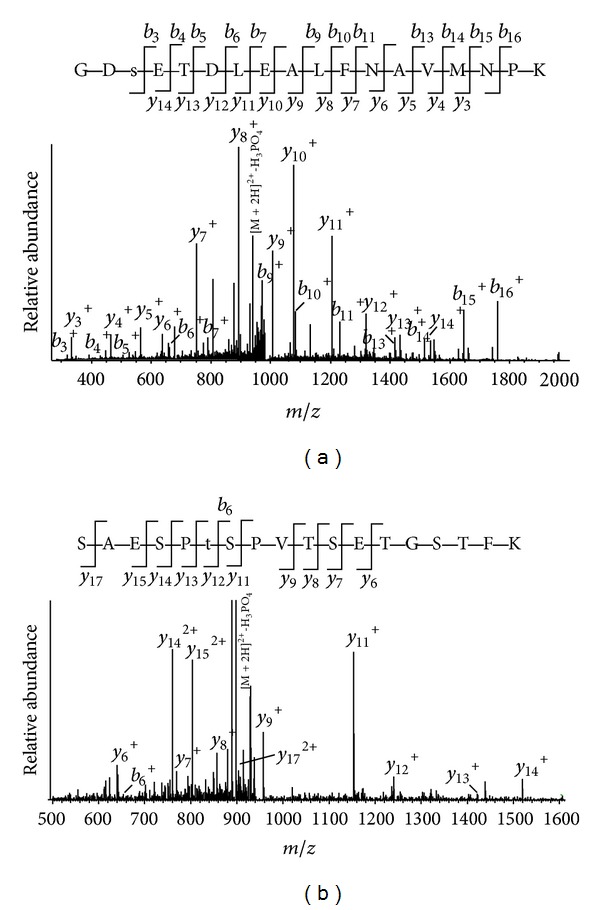
MS/MS spectra for identification of protein phosphorylation sites. (a) The MS/MS spectrum of a doubly charged peptide ion at* m/z* 1015.94 for MH_2_^2+^ corresponding to the mass of the peptide GDSETDLEALFNAVMNPK from YAP1. (b) The MS/MS spectrum of a doubly charged peptide ion at* m/z* 946.91 for MH_2_^2+^ corresponding to the mass of the peptide SAESPTSPVTSETGSTFK from Isoform 1 of A-kinase anchor protein 12. The underlined residues are the phosphorylation sites.

**Figure 3:**
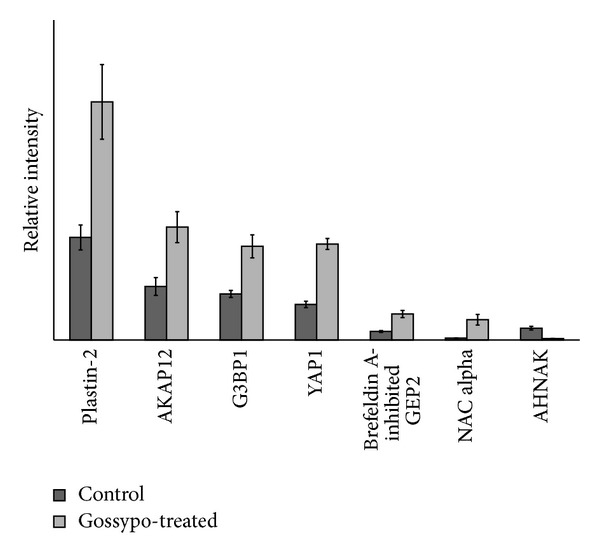
The relative abundance and fold changes of Isoform 1 of A-kinase anchor protein 12 (AKAP12), Plastin-2, Brefeldin A-inhibited guanine nucleotide-exchange protein 2, YAP1, Nascent polypeptide-associated complex subunit alpha, Ras GTPase-activating protein-binding protein 1 (G3BP1), and neuroblast differentiation-associated protein (AHNAK) from the untreated and gossypol-treated HOC1a cells. Cells treated with DMSO were used as the control.

The MS/MS spectrum of each identified phosphopeptide was manually confirmed. For example, the MS/MS spectra matched to phosphopeptides of YAP1 and Isoform 1 of A-kinase anchor protein 12 are displayed in Figure 2, respectively. In Figures 2(a) and 2(b), a loss of phosphate group (−98) was observed for both peptides indicating that peptides contain phosphorylated Ser or Thr residues. By matching b and y ions of the phosphorylated peptides, we identified that the underlined Ser residue in GDSETDLEALFNAVMNPK was phosphorylated in YAP1 (Figure 2(a)); so was the underlined Thr-6 residue in SAESPTSPVTSETGSTFK in AKAP12 (Figure 2(b)). Using the same score cutoff filter, 63 phosphoproteins were identified in three repeated experiments from gossypol-treated cells (Supplementary Table 3). In comparison to phosphorylated proteins identified in control, phosphorylation of 29 proteins changes upon gossypol treatment (Table 1). Using a label-free quantitation method, we semiquantified the fold change of phosphopeptides for 7 proteins (Figure 3). Intensities of phosphopeptides from Isoform 1 of A-kinase anchor protein 12 (AKAP12), Plastin-2, Brefeldin A-inhibited guanine nucleotide-exchange protein 2, YAP1, Nascent polypeptide-associated complex subunit alpha, and Ras GTPase-activating protein-binding protein 1 (G3BP1) increase while that of neuroblast differentiation-associated protein (AHNAK) decreases after gossypol treatment.

**Figure 4:**
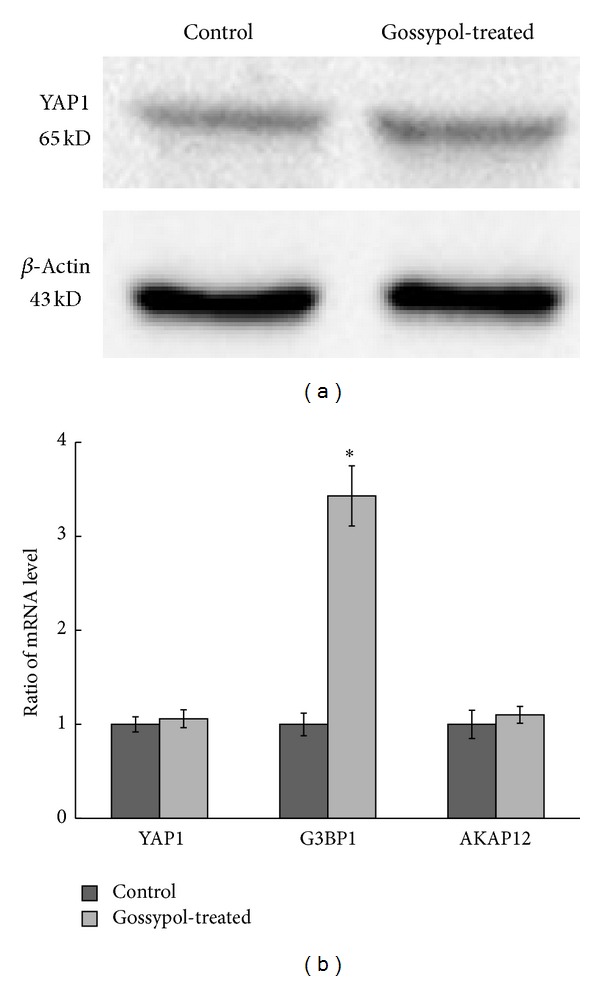
(a) Western blot showed that gossypol treatment does not induce upregulation of YAP1. Cells treated with DMSO were used as the control, and actin was used as protein loading control. (b) qPCR analysis of expressions levels of YAP1, AKAP12, and G3BP1. ^∗^ represents *P* < 0.05. Cells treated with DMSO were used as the control.

**Figure 5:**
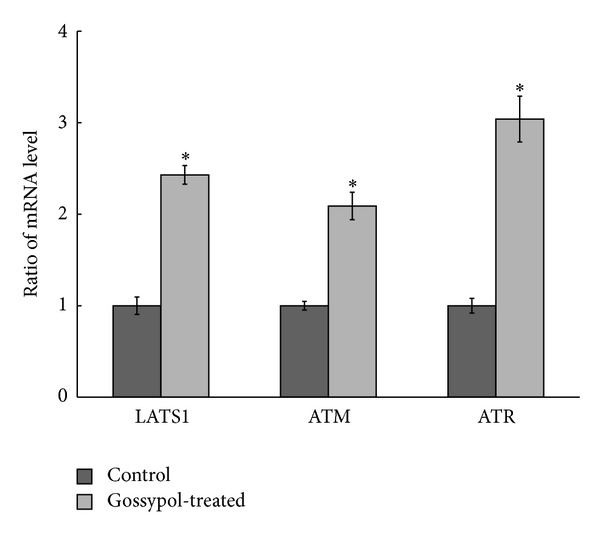
qPCR analysis of expressions levels of LATS1, ATM, and ATR. ^∗^ represents *P* < 0.05. Cells treated with DMSO were used as the control.

To examine whether phosphorylation changes are due to expression changes of the respective proteins, western blot and qPCR were used to quantify protein expressions and levels of mRNA in selected proteins. Western blotting of YAP1 (Figure 4(a)) showed that gossypol treatment does not induce upregulation of YAP1. This is also confirmed by qPCR analysis of YAP1 (Figure 4(b)). Data showed that the mRNA level of AKAP12 does not change, but that of G3BP1 increases upon gossypol treatment. To explore if the increased phosphorylation in YAP1, AKAP12, and Plastin is associated with their upstream regulators, we have also employed qPCR to quantify the mRNA expressions of LATS1, ATM, and ATR, showing that gossypol induced upregulation of LATS1, ATM, and ATR (Figure 5).

## 4. Discussion

Gossypol is one of the anticancer drugs in development. Studies have shown that gossypol targets a complex spectrum of proteins including Bcl-2 and signaling pathways. In the present study, we show that gossypol effectively induces apoptosis in ovarian cancer cells. Using a phosphoproteomic approach, we identified that gossypol treatment induced changes in phosphorylation of proteins associated with YAP1-Hippo pathway and cytoskeletal organization. To the best of our knowledge, this is the first report suggesting that gossypol-induced apoptosis is associated with phosphorylation of YAP1 and other actin-binding proteins.

Using HOC1a cells as a model system for ovarian cancer, we found that 42% of HOC1a cells went into apoptosis in response to 60 *μ*M gossypol treatment for 24 hours. We carried out a phosphoproteomic analysis to compare phosphoproteins between the untreated and gossypol-treated HOC1a cells. We identified about 9700 phosphopeptides at 1% false discovery rate. We only chose 62 phosphoproteins that were identified in three repeated experiments with higher scores (Supplementary Table 2) and their phosphorylation sites were confirmed by Scaffold PTM-Proteome Software and PhosphoRS that is an algorithm to determine individual site probabilities for each putative phosphorylation site. Using the same filter settings, we also identified 63 phosphoproteins from gossypol-treated cells, in which phosphorylation of 29 proteins changes with gossypol treatment as listed in Table 1. Using label-free quantitation, we analyzed the fold changes of phosphopeptides from 7 proteins (Figure 3): AKAP12, Plastin-2, Brefeldin A-inhibited guanine nucleotide-exchange protein 2, YAP1, Nascent polypeptide-associated complex subunit alpha, G3BP1, and AHNAK.

YAP1 is the transcriptional coactivator in the Hippo signaling pathway and is an oncogene in ovarian cancer by enhancing the transformed phenotype of ovarian cancer cell lines and conferring resistance to chemotherapeutic agents that are commonly used to treat ovarian cancer [45–48]. The high nuclear YAP expression was also found to be correlated with poor patient prognosis in the invasive epithelial ovarian cancer samples. This is because nuclear YAP is associated with regulation of cellular genes important for cell proliferation, cell death, cell migration, and epithelial mesenchymal transition. YAP proteins are phosphorylated by LATS kinases on multiple sites in the Hippo pathway. The phosphorylation of YAP resulted in repressing YAP activity by retention of YAP in the cytoplasm and targeting YAP protein for ubiquitin-mediated proteolysis. YAP1 contains five LATS-targeted consensus phosphorylation sequences (Hx[HRK]xx[ST]) at positions 61, 109, 127, 164, and 397 [49]. We identified phosphorylation at Ser 61 (Figure 2(a)) that increases upon gossypol treatment.

By western blot and qPCR analysis, the expression of YAP1 was found to remain unchanged (Figure 4). However, qPCR analysis showed that the mRNA level of LATS increased with gossypol treatment (Figure 4). This indicates that gossypol-induced upregulation of LATS targets YAP1 phosphorylation at Ser 61 residue in HOC1a cells, and, in turn, an increase of YAP phosphorylation leads to retention of YAP1 in cytoplasm to reduce cell proliferation. A recent study also revealed that G-protein-coupled receptors act upstream of the transcriptional coactivators YAP/TAZ and demonstrated that the Hippo signaling pathway regulates the actin cytoskeletal organization [50]. Therefore, we hypothesize that alternation of YAP1 phosphorylation contributes to gossypol-induced cell death through retention of YAP1 in cytoplasm and/or changes of the actin cytoskeletal organization.

Gossypol also induced phosphorylation changes in several actin-binding proteins including Src substrate cortactin, AHNAK, Plastin, and AKAP12. AHNAK is a giant propeller-like protein that translocates from cytosol to the plasma membrane to form a multimeric complex with actin and annexin 2/S100A10. Cortactin is also a cytoskeletal protein involved in coordinating actin reorganization during cell movement and its amino-terminal acidic domain binds to Arp2/3 and WASP complex at F-actin branches. Plastin is an actin-binding protein and is involved in actin filament bundle formation to regulate intracellular protein transport. Moreover, AKAP12 is a scaffold protein that contains 3 conserved PKA binding motifs to assist formation of actin-based cytoskeletal architecture. Studies have shown that AKAP12 is a tumor suppressor and reexpression of AKAP12 inhibits progression and metastasis of colorectal carcinoma [51–53]. Phosphoproteomic analysis has shown that AKAP12 and Plastin are substrates of ATM/ATR kinase [50]. By qPCR analysis, we found that gossypol induces upregulation of ATM and ATR gene expressions, indicating that gossypol induces upregulation of ATM to increase phosphorylation of AKAP12. Although we have not identified the ATM/ATR-targeted phosphorylation sites in AKAP12 and Plastin, our data suggest that phosphorylation changes of these actin-binding proteins affect the actin cytoskeletal organization and play a role in gossypol-induced cell death [54].

Taken together, we showed that gossypol induces apoptosis of ovarian cancer cells. Proteomic analysis of untreated and gossypol-treated cells reveals that gossypol induces phosphorylation changes in 29 proteins including YAP1 and AKAP12. Our results suggest that gossypol targets the actin cytoskeletal organization to induce ovarian cancer cell death through changing phosphorylation states of actin-binding proteins and activating YAP1-associated Hippo pathway.

## Supplementary Material

Supplementary Figure 1. Functional classification of identified phosphoproteins with PANTHER (http://www.pantherdb.org/)
Supplementary Table 1. Primers used for qPCR analysis in this work.
Supplementary Table 2. The phosphoproteins and phosphopeptides identified in ovarian cancer cell line, HOC1a.
Supplementary Table 3. The phosphoproteins and phosphopeptides identified in HOC1a cells treated with 10*μ*M Gossypol for 4 hours.

## Figures and Tables

**Table 1:** Gossypol-induced changes of phosphoproteins in HOC1a cells.

IPI accession	Protein name	Peptide sequence	Control	Gossypol-treated
IPI00002186	Brefeldin A-inhibited guanine nucleotide-exchange protein 2	GSSLSGTDDGAQEVVKDILEDVVTSAIK	+	++
IPI00008529	60S acidic ribosomal protein P2	KEESEESDDDMGFGLFD		+
IPI00237884	Isoform 1 of A-kinase anchor protein 12	SAESPTSPVTSETGSTFK	+	++
IPI00185919	Isoform 1 of La-related protein 1	GLSASLPDLDSENWIEVK		+
IPI00015953	Isoform 1 of nucleolar RNA helicase 2	NEEPSEEEIDAPKPK		+
IPI00102752	Isoform 1 of putative RNA-binding protein 15	SLSPGGAALGYR		+
IPI00451401	Isoform 2 of triosephosphate isomerase	KQSLGELIGTLNAAK		+
IPI00293312	Isoform 2 of zinc finger CCCH domain-containing protein 18	LGVSVSPSR		+
IPI00023748	Nascent polypeptide-associated complex subunit alpha	VQGEAVSNIQENTQTPTVQEESEEEEVDETGVEVK	+	++
IPI00604756	Nuclear receptor-binding protein	TPTPEPAEVETR		+
IPI00010471	Plastin-2	GSVSDEEMMELR	+	++
IPI00015029	Prostaglandin E synthase 3	DWEDDSDEDMSNFDR		+
IPI00012442	Ras GTPase-activating protein-binding protein 1	SSSPAPADIAQTVQEDLR	+	++
IPI00008708	Ribosomal L1 domain-containing protein 1	ATNESEDEIPQLVPIGK		+
IPI00029601	Src substrate cortactin	LPSSPVYEDAASFK; TQTPPVSPAPQPTEER; TQTPPVSPAPQPTEER		+
IPI00045051	Transcriptional activator protein Pur-beta	DSLGDFIEHYAQLGPSSPEQLAAGAEEGGGPR		+
IPI00009326	Yes-associated protein beta	GDSETDLEALFNAVMNPK	+	++
IPI00844578	ATP-dependent RNA helicase A	SEEVPAFGVASPPPLTDTPDTTANAEGDLPTTMGGPLPPHLALK	+	
IPI00010463	GTP-binding protein 1	GLGPPSPPAPPR	+	
IPI00009505	Isoform 1 of beta-2-syntrophin	GLGPPSPPAPPR	+	
IPI00216049	Isoform 1 of heterogeneous nuclear ribonucleoprotein K	METEQPEETFPNTETNGEFGK	+	
IPI00019848	Isoform 1 of host cell factor 1	SQCQTRQTSATSTTMTVMATGAPCSAGPLLGPSMAREPGGR	+	
IPI00782992	Isoform 1 of serine/arginine repetitive matrix protein 2	MALPPQEDATASPPR; THTTALAGRSPSPASGR	+	
IPI00026466	Isoform 2 of nipped-B-like protein	AITSLLGGGSPK	+	
IPI00150057	Isoform 2 of SWI/SNF complex subunit SMARCC2	DMDEPSPVPNVEEVTLPK	+	
IPI00165041	Isoform 6 of treacle protein	TSQVGAASAPAKESPR	+	
IPI00006122	Isoform MEA6 of cutaneous T-cell lymphoma-associated antigen 5	AFLSPPTLLEGPLR	+	
IPI00164672	mRNA-decapping enzyme 1A	HAPTYTIPLSPVLSPTLPAEAPTAQVPPSLPR	+	
IPI00103483	Negative elongation factor B	KPSPAQAAETPALELPLPSVPAPAPL	+	
IPI00021812	Neuroblast differentiation-associated protein AHNAK	FKAEAPLPSPK	++	+

^+^Identified phosphorylated proteins. ^++^More than 2-fold difference with respect to control.
